# Rapid Change in Articulatory Lip Movement Induced by Preceding Auditory Feedback during Production of Bilabial Plosives

**DOI:** 10.1371/journal.pone.0013866

**Published:** 2010-11-08

**Authors:** Takemi Mochida, Hiroaki Gomi, Makio Kashino

**Affiliations:** 1 Human and Information Science Laboratory, NTT Communication Science Laboratories, Nippon Telegraph and Telephone Corporation, Atsugi, Kanagawa, Japan; 2 Shimojo Implicit Brain Function Project, Exploratory Research for Advanced Technology, Japan Science and Technology Agency, Kawaguchi-shi, Saitama, Japan; 3 Department of Information Processing, Interdisciplinary Graduate School of Science and Engineering, Tokyo Institute of Technology, Tokyo, Japan; The University of Western Ontario, Canada

## Abstract

**Background:**

There has been plentiful evidence of kinesthetically induced rapid compensation for unanticipated perturbation in speech articulatory movements. However, the role of auditory information in stabilizing articulation has been little studied except for the control of voice fundamental frequency, voice amplitude and vowel formant frequencies. Although the influence of auditory information on the articulatory control process is evident in unintended speech errors caused by delayed auditory feedback, the direct and immediate effect of auditory alteration on the movements of articulators has not been clarified.

**Methodology/Principal Findings:**

This work examined whether temporal changes in the auditory feedback of bilabial plosives immediately affects the subsequent lip movement. We conducted experiments with an auditory feedback alteration system that enabled us to replace or block speech sounds in real time. Participants were asked to produce the syllable /pa/ repeatedly at a constant rate. During the repetition, normal auditory feedback was interrupted, and one of three pre-recorded syllables /pa/, /Φa/, or /pi/, spoken by the same participant, was presented once at a different timing from the anticipated production onset, while no feedback was presented for subsequent repetitions. Comparisons of the labial distance trajectories under altered and normal feedback conditions indicated that the movement quickened during the short period immediately after the alteration onset, when /pa/ was presented 50 ms before the expected timing. Such change was not significant under other feedback conditions we tested.

**Conclusions/Significance:**

The earlier articulation rapidly induced by the progressive auditory input suggests that a compensatory mechanism helps to maintain a constant speech rate by detecting errors between the internally predicted and actually provided auditory information associated with self movement. The timing- and context-dependent effects of feedback alteration suggest that the sensory error detection works in a temporally asymmetric window where acoustic features of the syllable to be produced may be coded.

## Introduction

During the development of speech production, different sorts of sensory feedback help to coordinate the movements of the respiratory, laryngeal, velopharyngeal, and articulatory subsystems. Cutaneous and/or somatosensory information on the status of multiple articulators and auditory information related to produced speech constitute important sources of feedback for speech motor control [1]. Various studies employing auditory feedback alteration have suggested that acoustic information is critical as regards learning and maintaining vowel production [2], [3] and voice pitch control [4], [5]. Evidence has also been obtained from humans and non-human primates showing that neural activity in the auditory cortex is modulated by self-produced vocalization [6], [7], [8], [9]. In concert with these studies, theoretical models of speech acquisition and production have been proposed, which hypothesize that speech targets represented in auditory space are achieved using an articulatory-to-auditory map trained on self-produced auditory feedback [10], [11]. However, debate continues as to whether such neural mechanisms also help to ensure stability in rapid and complex speech motor control [12], [13], aside from the well-studied reflexive adjustment of voice volume or pitch based on auditory information [5], [14], [15], [16], [17], [18]. Certain aspects of the effects of auditory feedback on speech articulation have been examined using the delayed auditory feedback (DAF) paradigm [19], [20], [21], [22], [23] where various types of speech disfluencies are induced, e.g., increased articulatory error, lengthened duration, augmented volume, and increased fundamental frequency. Similarly, a vocal duration reduction with an accelerated auditory feedback delay has also been reported [24]. However, the mechanisms that underlie these effects elicited by constant exposure to unusual feedback delay remain unclear. Auditory feedback may serve as an immediate source for the dynamic control of speech articulation, analogous to the well-known rapid adjustment of labial constriction based on cutaneous and/or somatosensory information [25], [26], [27], [28], [29].

In this study, we examined the online control mechanism for articulatory lip movement by suddenly shifting the auditory feedback timing in the ahead-of-time or delayed direction, and/or replacing the feedback syllable by other syllables, during the repetition of bilabial plosives /pa/. Labial distance trajectories under altered and normal feedback conditions were compared within a single cycle of lip closing/opening movement subsequent to the auditory alteration. Statistical analysis revealed that a quickened lip closing/opening movement was clearly elicited when the auditory feedback preceded the real production by 50 ms. On the other hand, such change was not significant when the feedback was provided more than 50 ms before the real production or was delayed, and/or when the feedback syllable was replaced by /Φa/ or /pi/. These results suggest (1) an underlying mechanism that detects errors between anticipated and actually provided auditory consequences for the rapid modification of subsequent movements, and (2) a temporally asymmetric window for detecting auditory errors in which acoustic features of the syllable to be produced may be coded.

## Materials and Methods

### Ethics Statement

All participants gave their written informed consent to participating in this study, which was approved by the Research Ethics Board of NTT Communication Science Laboratories.

### Participants

Ten adults (seven males and three females) aged from 21 to 39 participated in the experiments. All the participants were native speakers of Japanese and exhibited no obvious speech difficulties as judged by the experimenters.

### Apparatus

**Figure 1:**
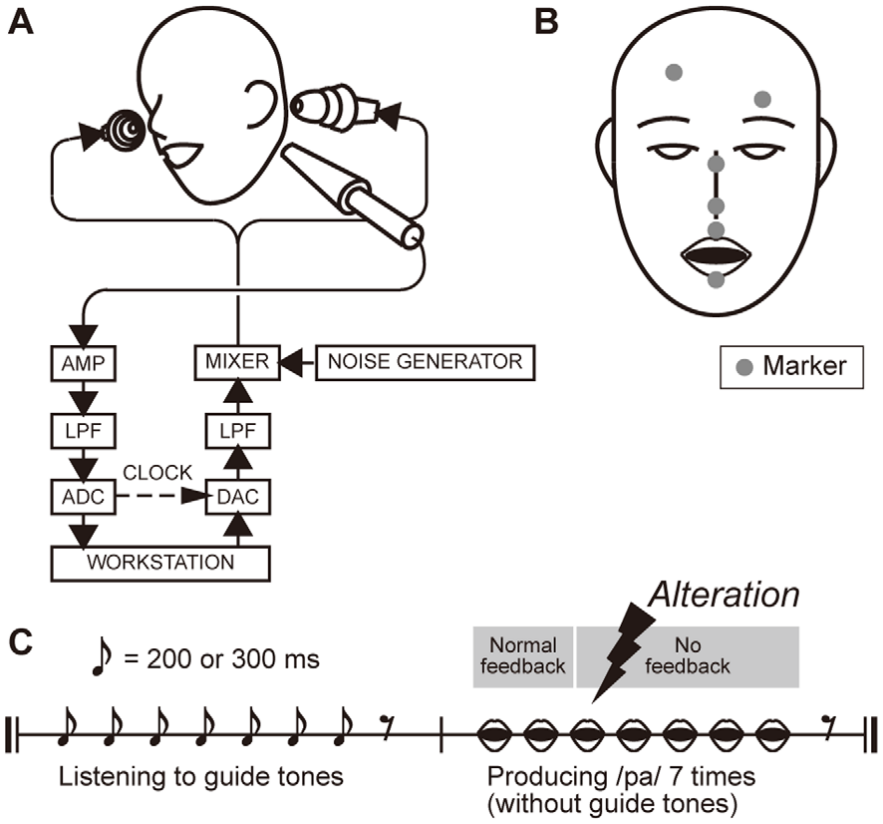
Experimental equipment and protocol. (**A**) Schematic diagram of auditory feedback alteration system. See text for details. (**B**) Placement of markers for measuring the three-dimensional motion of the upper and lower lips. Six markers were placed on the vermilion borders of the upper and lower lips in the midsagittal section, the bridge and tip of the nose, and the left and right side of the forehead. (**C**) Schematic diagram of experimental protocol. At the beginning of the trial, the participants heard a sequence of seven click tones with an interval of 200 or 300 ms through earphones. After hearing the final (seventh) click tone, the participants produced syllables at a rate identical to that indicated by the click tone sequence. No click tone was presented during the production period. Participants heard the unaltered speech feedback during the first two repetitions. The normal speech feedback was blocked after the second repetition, and /pa/, /Φa/, or /pi/ sound was presented once at −150, −100, −50, 0, +50, +100, or +150 ms from the predicted third repetition onset.

Figure 1A is a schematic diagram of the auditory feedback alteration system. The speech sounds produced by a participant are converted into voltage signals by an electret condenser microphone (Sony ECM-G3M driven by an Earthworks Microphone Preamp 1021). The signals are then filtered (NF 48 dB/oct filter P-85 in the phase-linear low pass mode) with a cutoff frequency of 6 kHz, and digitized at a sampling frequency of 16 kHz (Systems Design Service DASBOX Model-16/100). A custom made program for altering the input speech signals with a buffer size corresponding to 10 ms is run on a workstation. The processed signals are then converted to voltage signals (Systems Design Service DASBOX-16) and filtered (NF 48 dB/oct filter P-85 in the phase-linear low pass mode) with a cutoff frequency of 6 kHz. Finally, the voltage signals are converted into acoustic sounds and fed back to the participant bilaterally using earphones (Etymotic Research earphones ER-4S driven by Sony audio mixer SRP-X6004).

In the experiment, the participants sat on a chair and were asked to insert the earphones as deeply as possible in the ear canal. A microphone mounted in a floor stand was located close to the left ears of the participants who were asked to keep their heads in a fixed position throughout the experiments. The participants heard their own unaltered speech picked up by the microphone through the earphones while vocalizing an /a/ sound in their natural way. They were then asked to adjust the gain of the microphone so that they heard their own speech sounds most naturally. The participants were also asked to adjust the sound level of the pink noise they heard through the earphones, which was produced by a noise generator (Bruel & Kjaer Type 1405), while vocalizing an /a/ sound in their natural way, so that, as far as possible, they did not perceive their own bone-conducted auditory feedback, but without experiencing stress. The sound level of the noise chosen by the ten participants in the experiments was 61.5±2.75 dBSPL as measured by a probe microphone (Etymotic Research Probe Microphone ER-7C).

We chose an in-the-ear transducer with a view to eliminating the participants' own air-conducted auditory feedback most effectively. However, the occlusion effect caused by the in-ear earphone can influence the bone conduction threshold. The occlusion effect is the result of the acoustic energy created by the vibration of the walls of the external ear canal in response to a bone conducted signal trapped in the ear. When the tip of the earphone is fitted deeper in the ear canal, there is less opportunity for vibrations to occur and the occlusion effect is reduced [30]. This is why the participants were asked to insert the earphones as deeply as possible in the ear canal.

The three-dimensional motion of the upper and lower lips was measured with an optical motion capture system (Qualisys Qqus) at a sampling frequency of 250 Hz. Six low mass, retro-reflective markers with a diameter of 4 mm were placed on the vermilion borders of the upper and lower lips in the midsagittal section, the bridge and the tip of the nose, and the left and right side of the forehead, as shown in Fig. 1B. Two digital cameras placed on the left and right in front of the participant emitted infrared light that was reflected from the markers and back to the cameras. The position data of the four markers other than those on the upper and lower lips were used to calculate the relative positions of the lips with respect to the participant's head.

### Experimental procedures

In each trial in this experiment, the participants were asked to produce an isolated syllable /pa/ seven times while maintaining a constant speech rate. For each trial, the auditory feedback corresponding to the third repetition of /pa/ was altered by shifting the timing and/or replacing the type of syllable, while the subsequent feedback was blocked. A comparison of the articulatory lip movement under each altered condition with that under a normal condition enabled us to evaluate the effect of auditory feedback alteration on speech motor control more precisely than previous studies based on DAF. As for speech errors produced when employing DAF, their speech rate dependence can also be disputed in the light of certain controversial results [22], [23]. Therefore, two speaking rates (200 and 300 ms per syllable) were employed in our experiment in order to examine the speed dependence of the effect.

The experiment consisted of five test blocks and one control block. Each test block consisted of forty-six trials, where twenty-three different feedback conditions were employed for two different repetition rates (200 and 300 ms per syllable). Of the twenty-three feedback conditions, twenty-one were altered conditions where one of three syllables (/pa/, /Φa/, or /pi/) was presented at seven different timings (−150, −100, −50, 0, +50, +100, or +150 ms in relation to the onset of the third repetition), one was a blocked condition (no feedback after the second repetition), and one was unaltered. The control block consisted of twenty trials with unaltered feedback conditions, half of which were conducted at 200 ms per syllable and half at 300 ms per syllable.

In the experiment, the control block was introduced first, which took about 5 minutes, followed by five test blocks, each of which took about 10 minutes. There was a short break between each block. During the test blocks, the order of the feedback conditions applied to each participant was shuffled block by block. In the control block, the two syllable rates were alternated trial by trial.

### Tasks

Figure 1C depicts the trial protocol. At the beginning of the trial, the participants heard a sequence of seven guide click tones with a fixed interval of 200 or 300 ms through their earphones. After hearing the final (seventh) click tone, the participants were asked to produce syllables at a syllable rate identical to that indicated by the click tone sequence. No click tone was presented during the production period. As illustrated in Figure 1C, the participants heard unaltered speech feedback while producing the first two repetitions. The burst onset timing of the first two repetitions was detected by thresholding the segmental power of the signals calculated every 4 ms. The burst onset timing of the third repetition was predicted before it was produced, based on the interval between those of the first two repetitions. The normal speech feedback was blocked after the second repetition, and the sound /pa/, /Φa/, or /pi/, spoken by the corresponding participant, was presented once either at −150, −100, −50, 0, +50, +100, or +150 ms from the predicted third repetition onset. These sound stimuli /pa/, /Φa/, and /pi/ were recorded by the participants just before they undertook this task. Note that this method enabled us to investigate not only the effect of speech sound alteration, but also the effect of the early feedback of speech sound, which was impossible to examine using the previously employed online signal modification methods [17], [18], [19], [20].

**Figure 2:**
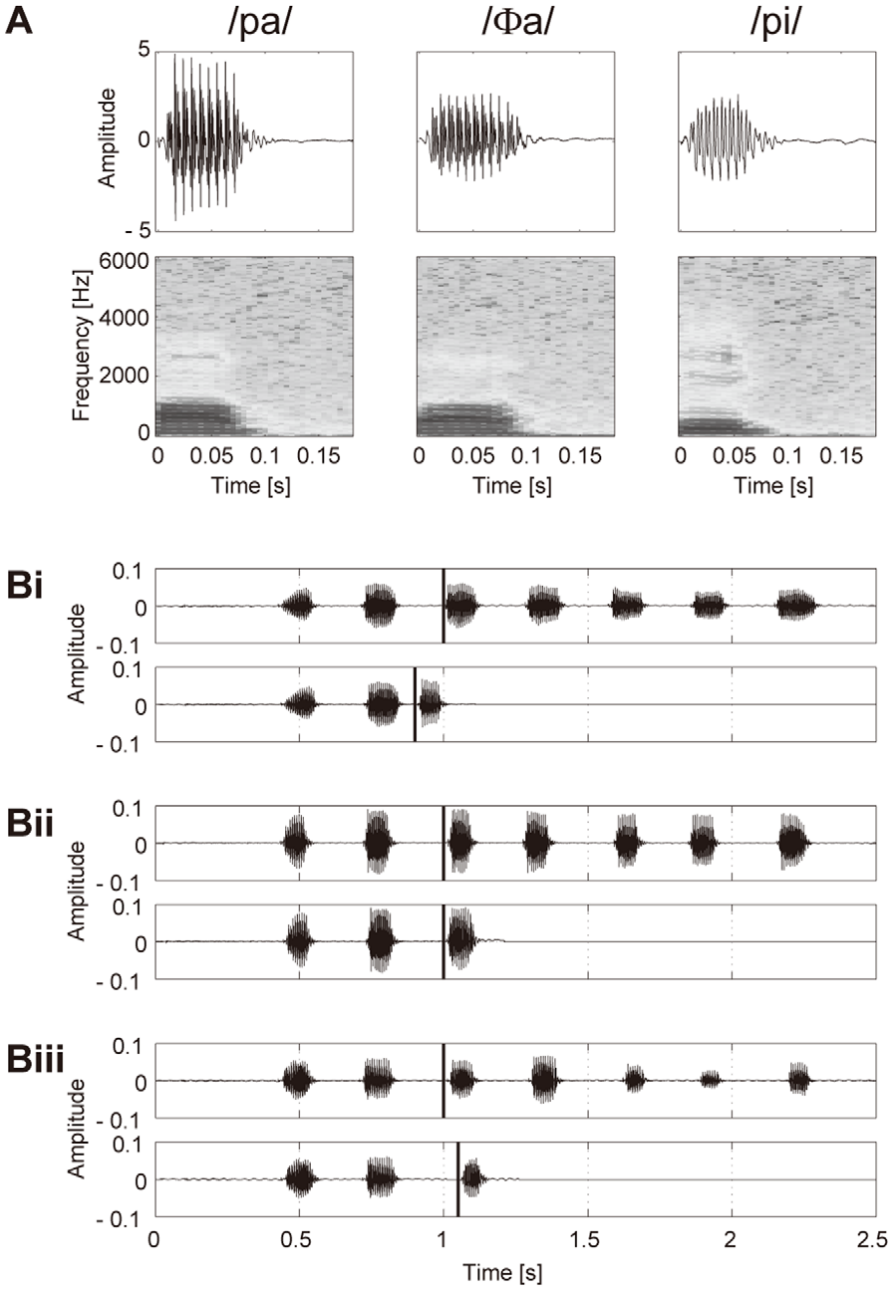
Examples of acoustic signals. (**A**) Examples of syllables stored for a participant when preparing sound stimuli. While the participant was producing /pa/, /Φa/, or /pi/ repeatedly, the burst onset timing of one syllable was detected in the same way as in the experiments, and 200 ms of the signals from the detected onset were stored while preserving the amplitude ratio among the syllables. When presenting these pre-recorded syllables in the experiments, the sound pressure level was adjusted by the computer program for every trial, based on that of the second repetition, so that the inter-syllabic ratio of the sound pressure level for /pa/, /Φa/, and /pi/ was maintained correctly as the participant produced the syllables in his or her natural way. (**B**) Examples of auditory feedback signals presented to a participant under three different conditions during the experiments, while he or she produced /pa/ seven times at a rate of 300 ms per syllable. In each pair of panels, Bi to Biii, the participant's speech signals are illustrated at the top, with the thick vertical line indicating the predicted onset of the third repetition. The corresponding auditory feedback signals are in the lower panels in Bi–Biii, with the thick vertical line indicating the onset of the altered auditory feedback signal. The auditory stimuli presented in Bi, Bii and Biii were /pa/ at −100 ms, /Φa/ at 0 ms and /pi/ at +50 ms from the predicted onset of the third repetition, respectively.

When preparing these stimuli, the participants repeated /pa/, /Φa/, and /pi/ in their most natural way. While the participants were producing these syllables, the burst onset timing of one syllable was detected in the same way as in the experiments, and 200 ms of the signals from the detected onset were stored for each of the three syllables, while preserving the amplitude ratio among the syllables. Examples of the stored syllables for a participant are shown in Fig. 2A. When these pre-recorded syllables were presented in the experiments, the sound pressure level was adjusted by the computer program in every trial, based on that of the second repetition, so that the inter-syllabic ratio of the sound pressure level for /pa/, /Φa/, and /pi/ was maintained correctly as each participant produced these syllables in his or her natural way.

Figure 2B shows examples of auditory feedback signals presented to a participant under three different conditions during the experiments, while repeating /pa/ seven times at a rate of 300 ms per syllable. In Figs. 2Bi–iii, the participant's speech signals are shown in the upper panel, where the thick vertical line indicates the predicted onset of the third repetition. The corresponding auditory feedback signals are shown in the lower panel, where the thick vertical line indicates the onset of the altered auditory feedback signal. The auditory stimuli presented in Figs. 2Bi–iii were /pa/ at −100 ms, /Φa/ at 0 ms and /pi/ at +50 ms from the predicted onset of the third repetition, respectively. The prediction error of the onset timing of the third repetition was at most 20 ms in the posthoc analyses of the results of trials performed under the unaltered auditory feedback condition.

### Data analysis

The time varying three-dimensional labial distance (LD) was calculated from the marker position data. For each participant, the LD trajectories of all trials were temporally aligned at the predicted third repetition onset by referring to the simultaneously recorded acoustic signals. The mean LD trajectory of five trials was obtained for each of forty-six different conditions in the five test blocks (twenty-three feedback types, two speech rates). The mean trajectory of ten trials from the control (normal feedback) block was also obtained for the two speech rates.

**Figure 3:**
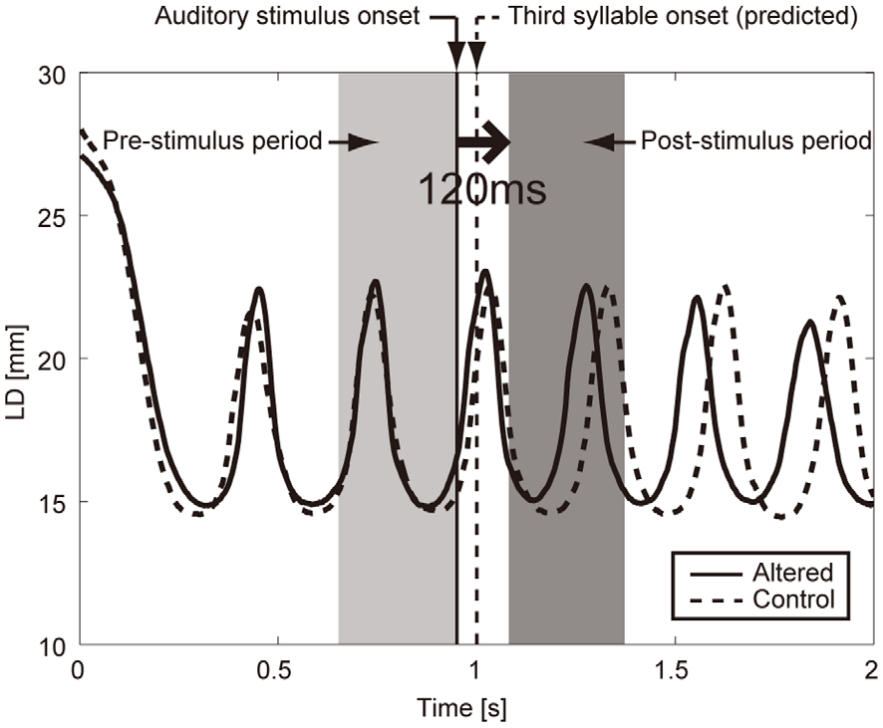
Definition of pre- and post-stimulus periods. The solid and dotted curves indicate the labial distance (LD) trajectories under the altered and control conditions, respectively. The thick vertical line indicates the onset timing of the auditory stimulus, while the dotted vertical line indicates the predicted third repetition onset. The bottom of curves within an opening-closing cycle corresponds to the instant of bilabial closure. The pre- and post-stimulus periods are highlighted by the light and dark gray rectangles, respectively. The lengths of the pre- and post-stimulus periods were identical to the syllable interval, i.e., 200 ms for a speech rate of 200 ms per syllable, and 300 ms for a speech rate of 300 ms per syllable. The top of the post-stimulus period was set at 120 ms after the onset timing of the auditory stimulus. The differences between two LD trajectories in each of the pre- and post-stimulus periods were calculated as the lags that provided the maximum cross-correlation between the two trajectories. The minus (plus) value of the lag corresponded to the ahead-of-time (delayed) shift of the trajectory caused by the auditory feedback alteration. See text for details.

The auditorily induced change in the labial movement was represented by a lag that provided the maximum cross-correlation between the LD trajectories under the altered and control conditions within the post-stimulus period. Note that this method was more stable and consistent than that using the displacement error or the velocity error, maybe because of the inter-participant variability in the time course of lip opening-closing cycle (see Figure S1). In Fig. 3, the solid and dotted curves in the figure indicate the mean LD trajectories under the altered and control conditions, respectively. (The bottoms of curves within an opening-closing cycle correspond to the instant of bilabial closure.) The thick vertical line indicates the onset timing of the auditory stimulus, while the dotted vertical line indicates the predicted third repetition onset. The beginning of the post-stimulus period was set at 120 ms after the stimulus onset, based on the fact that the short latency auditory-vocal response has a latency ranging from 100 to 150 ms [18]. A 200 (300) ms period was chosen for a speech rate of 200 (300) ms per syllable. The cross-correlation function 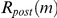 of the lag 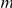 was represented by 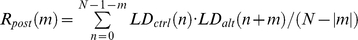, where 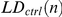 and 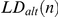 were the LDs at *n* under the control and altered conditions, respectively. Each LD trajectory was unbiased and windowed by a Blackman window to reduce the boundary effects. The lag that provided the maximum cross-correlation was represented as 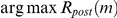. An ahead-of-time shift of the movement caused by an altered auditory feedback resulted in a minus lag value 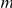, and vice versa.

To adjust for the phase difference between the trajectories of the altered and control conditions before alteration onset, the lag within the pre-stimulus period 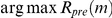 (also shown in Fig. 3) was calculated and subtracted from 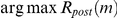. The pre-stimulus period was set at the same length as the post-stimulus period. The cross-correlation function 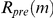 was calculated in the same way as 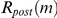.

## Results

### Labial distance trajectory

**Figure 4:**
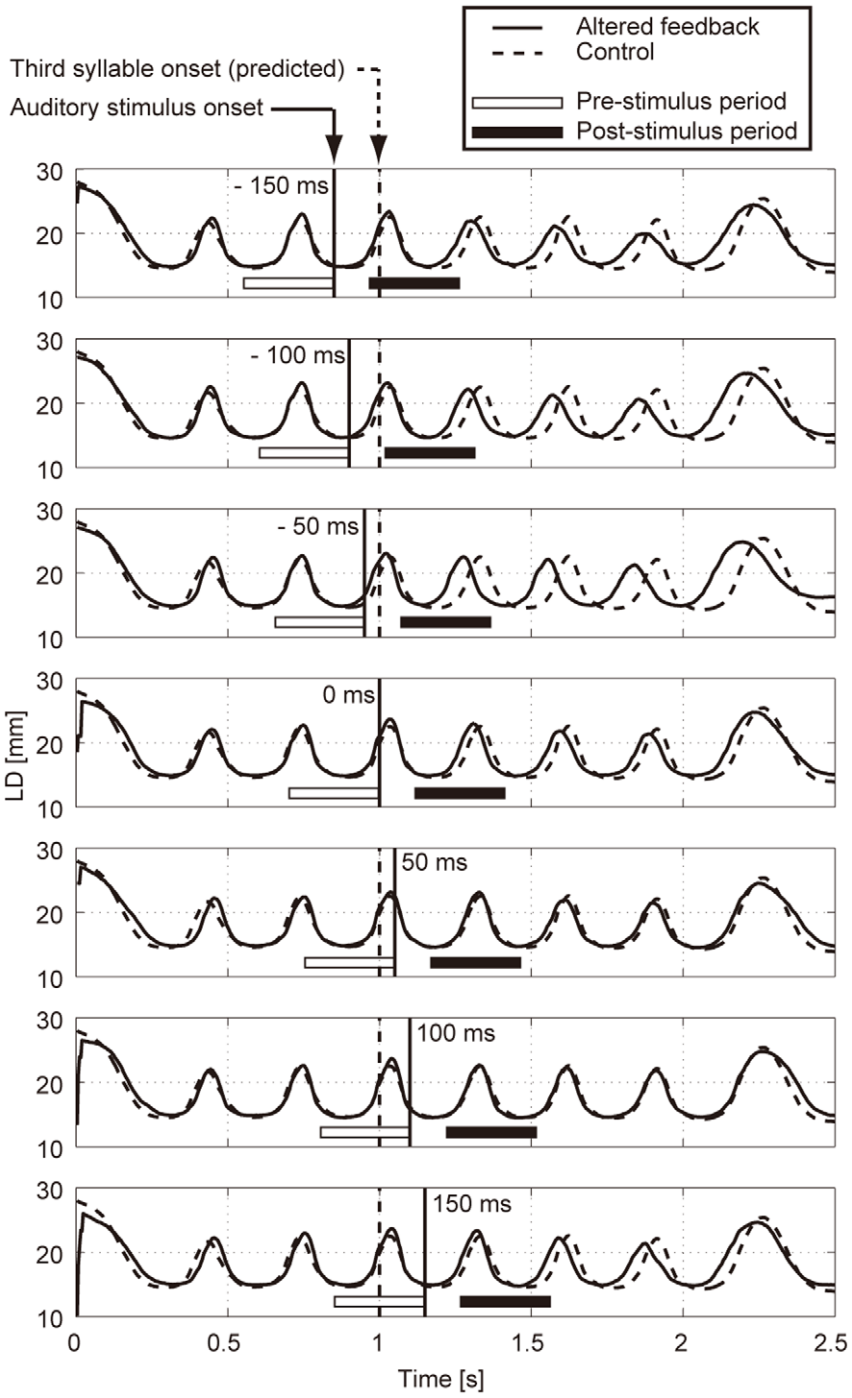
Labial distance (LD) trajectories of a participant while producing /pa/ at 300 ms per syllable. The auditory feedback conditions in each panel from the top to bottom were as follows: pre-recorded /pa/ was presented once at −150, −100, −50, 0, 50, 100, 150 ms from the predicted third repetition onset. The thick vertical line in each panel indicates the onset timing of the auditory stimulus, while the dotted vertical line indicates the predicted third repetition onset. The solid curve in each panel shows the mean LD trajectory of five trials over the test blocks. The mean trajectory of ten trials in the control (normal feedback condition) block is shown as a dotted curve. The white and black horizontal bars in each panel indicate the pre- and post-stimulus periods, respectively, for calculating the lag of the maximum cross-correlation between the LD trajectories under the altered and control conditions.

Figure 4 shows sample LD trajectory data during the production of /pa/ at a speech rate of 300 ms per syllable. The auditory feedback conditions shown from the top to bottom panels were as follows: pre-recorded /pa/ was presented once at −150, −100, −50, 0, 50, 100, 150 ms from the predicted third repetition onset. The solid vertical line in each panel indicates the onset timing of the auditory stimulus, while the dotted vertical line indicates the predicted third repetition onset. The solid curve in each panel shows the mean LD trajectory for five trials over the test blocks. The mean trajectory for ten trials in the control (normal feedback condition) block is shown as a dotted curve.

By comparing the two trajectories in each panel, the mouth opening movement subsequent to the auditory stimulus onset appeared generally to occur sooner for the −50 ms stimulus presentation. While a similar hasty movement was also observed for the −150 and −100 ms conditions, the effect seemed to be weaker. The deviation between the trajectories under each of the delayed feedback (50, 100, 150 ms) and control conditions was much smaller. Similar results were obtained for all ten participants.

In Fig. 4, the open and filled horizontal bars in each panel indicate the pre- and post-stimulus periods, respectively, for calculating the lag of the maximum cross-correlation between the LD trajectories under the altered and control conditions. The lag value may not necessarily reflect the exact amount of time shift, but will at least help to indicate which of the two series is leading the other, irrespective of which component of the amplitude, period, or phase of the LD trajectories was dominant in the difference. As observed in the top three panels in Fig. 4, the difference between the LD trajectories in the altered and control conditions tended to increase with time after the auditory alternation onset. Since such differences may be produced by a progressive accumulation of voluntary and involuntary effects, it would be difficult to specify the direct causal effect of auditory alteration on the LD trajectory. Therefore, in this study, we focused on the LD trajectory during a short period after the auditory alteration. The following subsection presents a statistical evaluation of the differences between LD trajectories under each of altered and control conditions.

### Auditorily induced rapid change in articulatory movement

**Figure 5:**
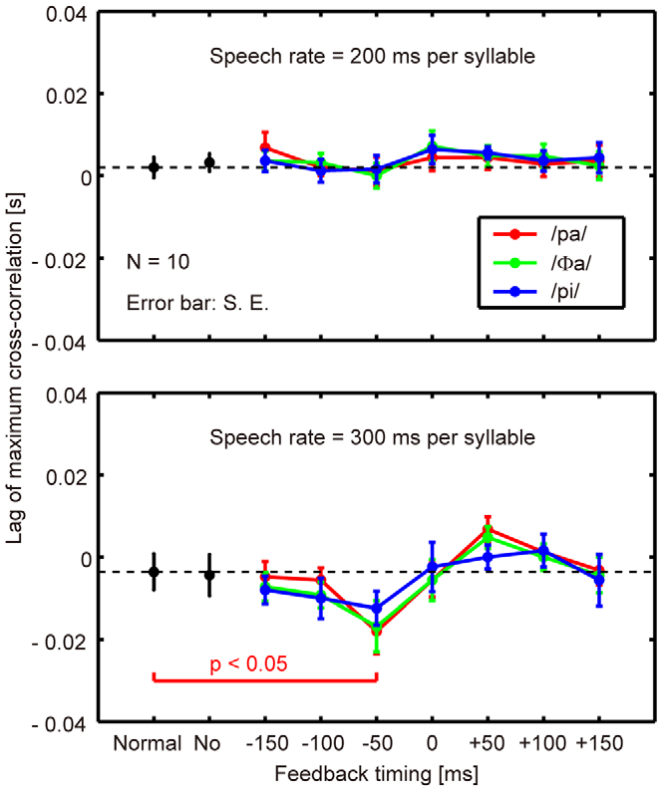
Lag of maximum cross-correlation (N = 10; error bar: standard error). Top: speech rate of 200 ms per syllable, bottom: speech rate of 300 ms per syllable. “Normal”: comparison of the normal feedback trials during the test blocks and those in the control block. “No”: the auditory feedback was interrupted after producing the second repetition. Other indices from “−150” through “+150” indicate the onset timing of the auditory stimulus against the predicted third repetition onset. The legends /pa/, /Φa/, and /pi/ show the syllable presented auditorily to the participants. The statistical difference between the values obtained under each altered feedback condition and those obtained under a “normal” condition was evaluated with a two-sided paired t-test.

Figure 5 shows the lag corresponding to the maximum cross-correlation (N = 10; error bar: standard error) between the LD trajectories under the altered and control conditions within the post-stimulus period, obtained by subtracting those within the pre-stimulus period. The minus value of the lag reflects an ahead-of-time shift of the articulatory lip movement compared with the control, and vice versa. The top and bottom panels show the results obtained when the speech rates were 200 and 300 ms per syllable, respectively. Each color indicates the syllable presented as a stimulus. “No” indicates a condition where no feedback was presented after the production of the second repetition. The condition indicated as “normal” refers to a comparison of the normal feedback trials during the test blocks and those in the control block, which reflects the variance in the baseline speech rate of each participant throughout the experiment.

For 22 altered conditions at each speech rate, the statistical significance of the difference from the “normal” condition was evaluated with a two-sided paired t-test (dF = 9 for all comparisons, with the Bonferroni adjustment). A statistically significant change (p<0.05) was found only when syllable /pa/ was presented 50 ms prior to the onset of syllable production for a rate of 300 ms per syllable. Under this condition, the auditory feedback alteration resulted in an ahead-of-time shift of the articulatory lip movement according to Fig. 5 (a minus lag value). A comparable large negative mean value was also found in Fig. 5 with a 50 ms preceding presentation of syllable /Φa/ at a rate of 300 ms per syllable. However, the difference from the normal condition was not statistically significant (p>0.05) owing to the variation across subjects. Also from Fig. 5, the maximum positive mean values were found for a 50 ms delayed presentation of syllables /pa/ and /Φa/ at a rate of 300 ms per syllable. However, these were also statistically insignificant (p>0.05). For a speech rate of 200 ms per syllable, the effects of auditory feedback alteration on the articulatory lip movement were found to be insignificant under all the conditions tested (p>0.05).

From the experimental results, we concluded that an ahead-of-time shift in the articulatory lip movement emerged rapidly when the auditory feedback preceded the real syllable production by 50 ms. However, too early a manipulation (−150 and −100 ms) of the auditory feedback did not significantly affect the subsequent articulatory lip movement. The delayed feedback also produced no significant change. Syllables that were not identical to those of the speech task (/Φa/ and /pi/) had no significant effect even when they were fed back 50 ms prior to the real syllable production. Finally, the articulatory changes were not significant for the faster speech rate (200 ms per syllable) under any of the alteration conditions tested.

## Discussion

### Time-asymmetric effect of auditory feedback alteration

The experimental results obtained in the current study showed that the ahead-of-time and delayed auditory feedback affected the articulatory lip movement in a time-asymmetric manner during repetitive syllable production. Significantly hastened articulation at around 120 ms from the auditory alteration onset occurred when the auditory stimulus was presented 50 ms prior to the onset of syllable production. Taken together with the hypothetical feedforward and feedback mechanisms of speech motor control [31], the hastened articulation could be regarded as a sort of rapid compensatory articulation in the time domain, which was induced by a sensory error caused by the progressive auditory input. However, the feedback alteration effect was not significant when the feedback timing was earlier (−150 and −100 ms). This fact seemed to suggest that an internal simulation of the auditory consequences of speech motor commands is not completed 100 ms prior to the initiation of the articulatory lip movement.

More interestingly, our experimental result revealed that no delayed feedback had a significant effect on the subsequent lip movement. One possible explanation for this result may be an imperfect masking of the air- and bone-conducted auditory feedback. In our experiment, an in-ear earphone was used to realize the effective isolation of the air-conducted feedback of the participants' own speech output. In addition, a masking noise was delivered to their ears to disturb the sensation and/or perception of the air- and bone-conducted feedback to a certain degree. However, even a small amount of natural feedback might still reduce the effect of sensory error on the motor control compared with ahead-of-time feedback alteration. This might result in the insufficient effect of the delayed auditory feedback.

Another possible mechanism for the temporally asymmetric effect could be related to the response attenuation in the auditory cortex resulting from self-produced vocalization [6], [7], [8], [9]. The precise temporal processing properties of such auditory attenuation on the time course of speech production, however, are less well understood. Further experimental and theoretical investigations are required to clarify the precise mechanisms underlying the time-asymmetric effect of auditory feedback alteration on the speech articulatory movement obtained in our experiment.

### Context dependence of auditorily-induced response

The experimental results showed that the auditory feedback of /Φa/ and /pi/ did not significantly change the subsequent lip movement, irrespective of the timing of the feedback. Taking this fact together with the hypothetical feedback-feedforward error correction mechanism [31], articulatory compensation in the time domain might be considered rather insensitive to an auditory input whose acoustic feature is not identical to that of the internal prediction.

**Figure 6:**
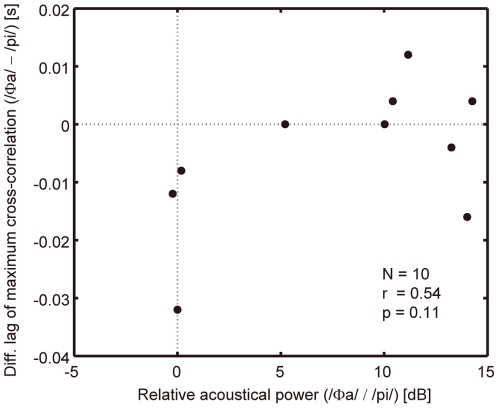
Magnitude difference in auditory-induced articulatory change against relative acoustical power of auditory feedback. The abscissa is the relative acoustical power between /Φa/ and /pi/. The ordinate is the difference between the mean lag shown in Fig. 5 for /Φa/ and /pi/ feedback 50 ms prior to the production onset under a rate of 300 ms per syllable. The correlation coefficient for ten participants was r = 0.54 (p = 0.11, dF = 8).

The results also revealed of the effect of /Φa/ had a larger mean value than that of /pi/ being fed back 50 ms prior to /pa/ production at a rate of 300 ms per syllable, though both were statistically insignificant. One suspected cause is that /pi/ has a smaller relative acoustical power than /Φa/. In the experiment, the auditory feedback amplitude of each syllable was dynamically adjusted so that its syllabic power ratio to the syllable /pa/ to be produced by each participant was matched with that in his/her natural production. (See the Task subsection for details.) Figure 6 shows the relationship between the relative syllabic power of the auditory feedback and the difference in the magnitude of auditorily-induced articulatory change on a participant-by-participant basis (N = 10). If the magnitude of the articulatory change were dependent on the power of the auditory feedback, the data in Fig. 6 would exhibit a negative correlation. However, the correlation coefficient for ten participants was found to be low (r = 0.54, p = 0.11, dF = 8). Therefore, the smaller mean value of the effect of /pi/ feedback did not appear to result from its smaller amplitude.

Another possible cause of the smaller mean effect of the /pi/ feedback could be related to a larger acoustic deviation of /pi/ from /pa/ compared with that of /Φa/, in the light of the evidence showing that the auditory cortex responded differently to self-produced and externally produced speech sounds during speech production [9]. The auditory input of /pi/ while producing /pa/ might not be processed as a self-produced sound because of the large difference in vowel quality between /a/ and /i/ such as the location of the formants, despite the invariant feature of the initial /p/ independent of the following vowel [32].

### Speech rate dependency of response

The experimental result showed that none of the altered auditory feedback tested under the faster speech condition (200 ms per syllable) induced significant articulatory changes. So far little has been reported about the dependence of the auditory alteration effect on speech rate. There have been conflicting results regarding the speech-rate dependence of DAF-induced disfluencies, where speech errors were found to decrease [23] or increase [22] as the speaking rate increased. Most of the speech errors both the above studies involved various suprasyllabic features, which may not be a direct consequence of the short-latency auditory-motor response as obtained in our experiment. Further investigation is required to untangle the sources of the complex speech errors induced by DAF, and to understand the mechanism underlying the speech-rate dependence of the auditory-motor response.

A study on the accuracy with which speakers repeat a monosyllable in time with an external rhythm suggested two underlying processes depending on the repetition rate [33]. At a rate of 1 to 3 times per second, speakers could compensate for a discrepancy in timing between a syllable and the external guide tone in an adjacent or neighboring utterance (“ongoing processing”), while at a rate of 4 to 6 times per second, such one-by-one processing did not work (“holistic processing”). Considering our experimental condition in the light of Hibi's work, a rate of 200 ms per syllable is classified as holistic processing where the one-by-one adjustment of utterances was impossible. On the other hand, a rate of 300 ms per syllable (equivalent to 3.3 times per second) can be classified as either ongoing or holistic processing. Such a difference in the underlying processing strategy might have caused the speech rate dependence of the auditory-motor response obtained in our experiment. However, the speech task used in our experiment was very different from that used in Hibi's work in that the participants were required to repeat the syllable in a self-paced manner with no external rhythm provided while speaking. Another processing mechanism may be involved in the self-paced rhythmic production.

### Language dependency of response

From the viewpoint of rhythmic properties, languages are considered to be classified as stress-, syllable-, or mora-timed, although a quantitative measure of speech rhythm has not been established. While the results of the current study were obtained from Japanese speakers, it would also be interesting to consider whether the same behavior occurs in other language speakers. Language-specific aspects of temporal organization of the kinematics of lower lip-jaw articulation have been compared between English, French, and Japanese, which are assumed to be examples of stress-, syllable-, and mora-timed languages, respectively [34]. They have used reiterant speech task in which speakers were required to replace each syllable of a target phrase with a single syllable such as /ba/ or /ma/, while trying to maintain the rhythmic character of the original [35]. They have found highly linear relation between peak velocity and displacement in lower lip movement for all three languages, and concluded that the dynamics could be modeled as a universal second-order system with language-specific parameter settings. It is therefore inferred that, as far as the repetitive syllable production task is concerned, the auditory-motor effect observed in the current study would be expected to occur also in speakers other than Japanese.

### Conclusion

A rapid auditorily induced change in articulatory lip movement was found when auditory feedback preceded real syllable production by 50 ms when isolated syllables were spoken repeatedly at a rate of 300 ms per syllable. The change was not significantly induced when the feedback occurred earlier than 50 ms or was delayed, and/or the feedback syllable was replaced by other syllables. The results suggested that a compensatory mechanism detected sensory errors between the internally predicted and actually provided auditory information associated with the self-produced speech, by using a temporally asymmetric window in which acoustic features of the syllable to be produced may be coded. This study provides evidence that the temporal dynamics of articulatory lip movement must be correctly maintained not only with somatosensory feedback resulting from peripheral motor activation but also with auditory feedback of self-produced speech.

## Supporting Information

Figure S1 Inter-participant variability in the time course of lip opening-closing behaviors. The solid and dotted curves indicate temporal patterns of the labial distance (left panels) and their first time-derivatives (right panels) under altered feedback and control conditions, respectively, during the repetitive production of /pa/ at a rate of 300 ms per syllable for each participant (P1 - 10). In the altered condition, the auditory feedback /pa/ was presented 50 ms prior to the predicted production onset. The thick vertical line indicates the onset timing of the auditory stimulus, and the dotted vertical line indicates the predicted third repetition onset. The pre- and post-stimulus periods used in the cross-correlation analysis are highlighted by the light and dark gray rectangles, respectively. As comparing the graphs of different participants, the displacement and its velocity patterns varied, and the timing of initiation of behavioral changes (e.g., temporal shift) by the feedback alteration were also different across the participants. Because of these variability, the kinetic variables such as the displacement and its time derivative were not useful to extract the common behavioral change across participants. In the main analysis, we therefore hired cross-correlation value as a lag-lead index because of robustness to the inter-participant behavioral variability.
(0.20 MB TIF)
