# Supplementary figures and images for: Rapid Change in Articulatory Lip Movement Induced by Preceding Auditory Feedback during Production of Bilabial Plosives
Source: PLoS One. 2010 Nov 8;5(11):e13866. doi: 10.1371/journal.pone.0013866 (PMC2975624; doi:10.1371/journal.pone.0013866)

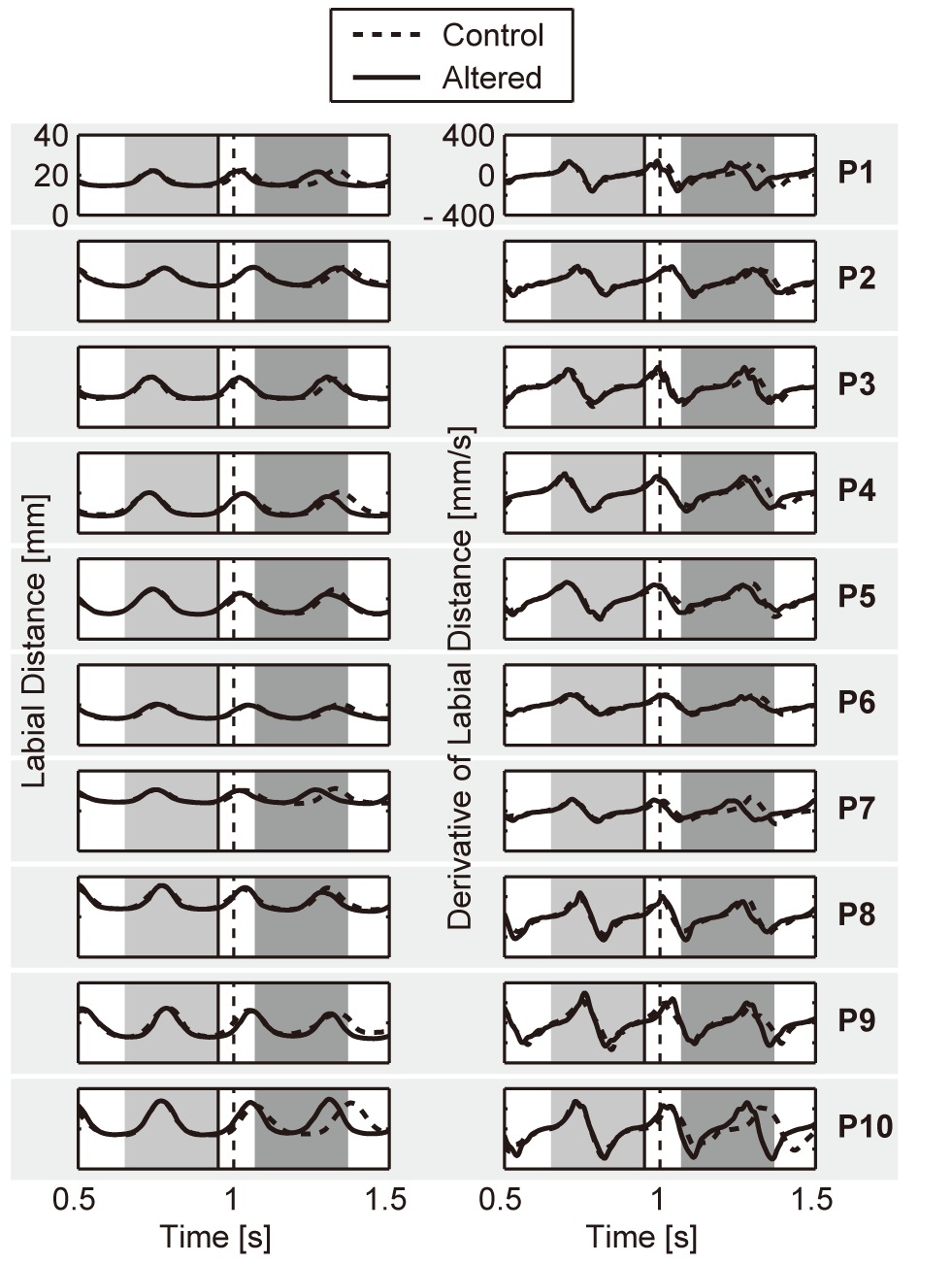

Supplement: Figure S1 — Inter-participant variability in the time course of lip opening-closing behaviors. The solid and dotted curves indicate temporal patterns of the labial distance (left panels) and their first time-derivatives (right panels) under altered feedback and control conditions, respectively, during the repetitive production of /pa/ at a rate of 300 ms per syllable for each participant (P1 - 10). In the altered condition, the auditory feedback /pa/ was presented 50 ms prior to the predicted production onset. The thick vertical line indicates the onset timing of the auditory stimulus, and the dotted vertical line indicates the predicted third repetition onset. The pre- and post-stimulus periods used in the cross-correlation analysis are highlighted by the light and dark gray rectangles, respectively. As comparing the graphs of different participants, the displacement and its velocity patterns varied, and the timing of initiation of behavioral changes (e.g., temporal shift) by the feedback alteration were also different across the participants. Because of these variability, the kinetic variables such as the displacement and its time derivative were not useful to extract the common behavioral change across participants. In the main analysis, we therefore hired cross-correlation value as a lag-lead index because of robustness to the inter-participant behavioral variability. (0.20 MB TIF) [file pone.0013866.s001.tif]
